# Integrated palliative care in the Spanish context: a systematic review of the literature

**DOI:** 10.1186/s12904-016-0120-9

**Published:** 2016-05-13

**Authors:** Eduardo Garralda, Jeroen Hasselaar, José Miguel Carrasco, Karen Van Beek, Naouma Siouta, Agnes Csikos, Johan Menten, Carlos Centeno

**Affiliations:** Atlantes Research Programme, Institute for Culture and Society, University of Navarra, Campus Universitario, 31009 Pamplona, Navarra Spain; Department of Anesthesiology, Pain and Palliative Medicine, Radboud University Nijmegen Medical Centre, Nijmegen, The Netherlands; Department of Radiation-Oncology and Palliative Medicine, University Hospital Gasthuisberg, Leuven, Belgium; Faculty of Medicine, Institute of Family Medicine, University of Pécs Medical School, Pécs, Hungary; Instituto de investigación sanitaria de Navarra (IdiSNA), Pamplona, Navarra Spain

**Keywords:** Integration, Palliative Care, Spain, Model, Guideline, Pathway

## Abstract

**Background:**

Integrated palliative care (IPC) involves bringing together administrative, organisational, clinical and service aspects in order to achieve continuity of care between all actors involved in the care network of patients receiving palliative care (PC) services. The purpose of this study is to identify literature on IPC in the Spanish context, either in cancer or other advanced chronic diseases.

**Methods:**

Systematic review of the literature about IPC published in Spain between 1995 and 2013. Sources searched included PubMed, Cochrane Library, Cinahl, the national palliative care Journal (Medicina Paliativa), and Google. Evidence on IPC in care models, pathways, guidelines and other relevant documents were searched. Additionally, data were included from expert sources. Elements of IPC were considered based on the definition of IPC and the Emmanuel´s IPC tool. The main inclusion criterion was a comprehensive description of PC integration.

**Results:**

Out of a total of 2,416 titles screened, 49 were included. We found two models describing IPC interventions achieving continuity and appropriateness of care as a result, 12 guidelines or pathways (most of them with a general approach including cancer and non-cancer and showing a theoretical IPC inclusion as measured by Emmanuel’s tool) and 35 other significant documents as for their context relevance (17 health strategy documents, 14 analytical studies and 4 descriptive documents). These last documents comprised respectively: regional and national plans with an IPC inclusion evidence, studies focused on IPC into primary care and resource utilisation; and descriptions of fruitful collaboration programmes between PC teams and oncology departments.

**Conclusions:**

The results show that explications of IPC in the Spanish literature exist, but that there is insufficient evidence of its impact in clinical practice. This review may be of interest for Spanish-speaking countries and for others seeking to know the status of IPC in the literature in their home nations.

**Electronic supplementary material:**

The online version of this article (doi:10.1186/s12904-016-0120-9) contains supplementary material, which is available to authorized users.

## Background

The European Union is experiencing an acute ageing of the population in recent decades with growing numbers of patients suffering from cancer and non-cancer disease [1]. Spain is also confronted with this development and has to develop palliative care (PC) services in response, aiming to improve the quality of life for patients and their families facing life-threatening illness.

In order to handle complex care situations, a sustained, expert and quality care provision is needed. Continuity of care is essential for patients with complex needs and engagement with a variety of service providers. In order to achieve this, it is necessary to integrate PC at all care levels and across interprofessional team and agency boundaries [2].

The challenge of integrating PC into the health system, at different care levels, for cancer and non-cancer, has already been acknowledged in the PC Strategy of the 2007 National Health System of Spain. It was estimated that 380.000 people die in Spain every year and that between 50 and 60 % of these may need PC in the last stage of their illness. The Strategy clearly states the need for coordinated action between diverse health providers to guarantee continuity of care, the timely identification of patients with PC needs, evidence of care planning that addresses the particular needs of patient and family caregivers, the provision of appropriate services, and on-going assessment systems [3].

This paper attempts to identify integration of PC in administrative, organisational, health services and clinical documents in the Spanish literature, either in cancer or other advanced chronic disease.

## Methods

A systematic review following PRISMA guidance [4] was conducted including scientific and grey literature. IPC has been defined as the administrative, organisational, clinical and service aspects in order to achieve continuity of care between all actors involved in the care network of patients receiving PC [5].

### Search strategy

Five different sources were searched: 1) PubMed database, including MeSH and free text terms (Table 1); 2) The Cochrane library database (Table 2); 3) The Cinahl database (Table 3); 4) The only PC Spanish journal “Medicina Paliativa” (“Palliative Medicine”) (manually searched); 5) Google (www.google.com) with (Table 4); and 5) Experts from different national Spanish societies of medicine (neurology, pulmonology, nephrology, geriatrics, cardiology and medical oncology,) were sent a letter to recommend experts on integrated care that could suggest studies on the topic in Spain.

**Table 1:** PUBMED search terms and chain

(Spain[MeSH Terms]) AND (((hospices OR supportive care OR supportive care OR end of life care OR palliative OR palliative care [MeSH Terms] OR hospice* OR terminal care OR coordinated care OR integrated care OR transmural care OR progressive patient care) AND (“end stage disease” OR end stage disease* OR dying OR death [MeSH Terms] OR Chronic disease [MeSH Terms] OR Chronic disease* OR terminally ill* OR terminally ill [MeSH Terms] OR cancer) AND (care pathway* OR care pathway OR pathway* OR patient transfer* OR patient transfer OR patient care team OR managed care program* OR continuity of patient care OR patient care management OR patient care plan* OR patient care planning OR illness trajectory OR “advanced care planning” OR advanced care planning OR delivery of health care OR models of care OR model of care OR model organizational OR models organizational OR organizational model* OR guideline*) NOT ((birth) OR child) OR pediatrics)) NOT ((animals[mh] NOT humans[mh])))

**Table 2:** COCHRANE library search terms and chain

((palliative medicine) or (palliative care)) and ((guideline) or (pathway) or (model) or (plan) or (programme))and ((integrate) or (integrated) or (integrative) or (integration)) and (Spain)	
((medicina paliativa) or (cuidados paliativos)) and ((guía) or (vía) or (modelo) or (plan) or (programa))and ((integrar) or (integrado) or (integrativo) or (integración)) and (España)

**Table 3:** CINAHL database search terms and chain

((palliative medicine) or (palliative care)) and ((guideline) or (pathway) or (model) or (plan) or (programme))and ((integrate) or (integrated) or (integrative) or (integration)) and (Spain)	
((medicina paliativa) or (cuidados paliativos)) and ((guía) or (vía) or (modelo) or (plan) or (programa))and ((integrar) or (integrado) or (integrativo) or (integración)) and (España)

**Table 4:** GOOGLE search terms and chain

guía paliativos OR programa oncología OR cáncer OR neurología OR neumología OR nefrología OR cardiología OR respiratoria OR cardíaca OR renal OR neurológica OR demencia “paliativo OR terminal” filetype:pdf	

### Search criteria

The search period dated from January 1995 (based on the publication year of the Calman-Hine report [6] which constitutes the first national cancer plan in Europe) to December 2013. Documents regarding interventions aimed at children, integrated mechanisms only focusing on the terminal phase (imminent death) and opinion in clinical case reports and editorial letters were excluded.

### Data selection

We concentrated on evidence addressing models, guidelines and pathways in IPC in cancer and chronic advanced disease. Models were considered to be “project models” implemented in a particular setting and describing an effective integration of PC; guidelines were those defined by the AGREE instrument as systematically developed statements to assist practitioner and patient decisions about appropriate health care for specific clinical circumstances [7]; and care pathways were considered to be complex interventions designed for mutual decision making and the organisation of care processes for a well-defined group of patients during a well-defined period [8].

Secondly, we searched documents related to strategy or of description and evidence of IPC. These included strategic documents (National or Regional public plans promoting and pointing out the need for IPC), descriptive documents (theoretical integrative programmes, models and general situation of PC) and analytical studies (observational or experimental studies assessing an experience or IPC intervention but different to models in terms of their level of effectiveness and their score in the Hawker et al. tool [9]).

All identified documents were downloaded into a database including the title, abstract and summary or introduction (depending on the type of document). Two researchers separately selected material for the data extraction phase (disparities were resolved by consensus). Only selected documents were screened for full text revision and data extraction.

### Data extraction

The data extraction form included: title, authors and collaborators, type of document (model, guideline, pathway or other), date, setting, and type of disease, participants’ demographics, study design, intervention, setting, outcome measures, results and quality assessment according to Hawker et al. [9]. The protocol of the Hawker et al. appraisal tool [9] rates the following aspects from 1 (very poor) to 4 (good): “1) Abstract and title: Did they provide a clear description of the study?; 2) Introduction and aims: Was there a good background and clear statement of the aims of the research?; 3) Method and data: Is the method appropriate and clearly explained?; 4) Sampling: Was the sampling strategy appropriate to address the aims?; 5) Data analysis: Was the description of the data analysis sufficiently rigorous?; 6) Ethics and bias: Have ethical issues been addressed, and what has necessary ethical approval gained? Has the relationship between researchers and participants been adequately considered? 7) Results: Is there a clear statement of the findings?; 8) Transferability or generalizability: Are the findings of this study transferable (generalizable) to a wider population?; 9) Implications and usefulness: How important are these findings to policy and practice?”

### Content assessment

Specific information relating to pathways and guidelines such as description of the document, inflection point (prognosis), and the presence of a separate reference to another guideline or pathway, were included in the database. Studies were included that met two or more of Emmanuel’s criteria as agreed by the InSup-C Consortium for its completeness of the IPC content [10]. This tool is comprised of different indicators rating the level of PC integration and explaining how is it being integrated. It proposes 11 specific criteria: “discussion of illness limitations and prognosis; recommendations for conducting a whole patient assessment including the patient’s physical, social, psychological and spiritual issues, their family and community setting; recommendations for when to review assessments; recommendations for when PC should be integrated; assessment of the patient’s goals for care; continuous goal adjustment as the illness and the person’s disease progresses; PC interventions to reduce suffering; advance care planning; recommendations on involving a PC team; recommendations on PC at the last moments of life; and recommendations on grief and bereavement.”

### Quality assessment

A quality assessment process agreed by the project research team was made to determine the quality of the guidelines and pathways according to the manner in which they were developed: systematic review, consensus methods, evidence based and quality assessment; systematic review and consensus methods; systematic review only; consensus methods only; unclear methods; and other options. Data from descriptive and analytic studies also underwent a quality evaluation using the Hawker et al. critical appraisal tool [9].

### Ethical approval

Ethical approval was not required as all the information is already published and human beings were not involved.

## Results

**Fig. 1:**
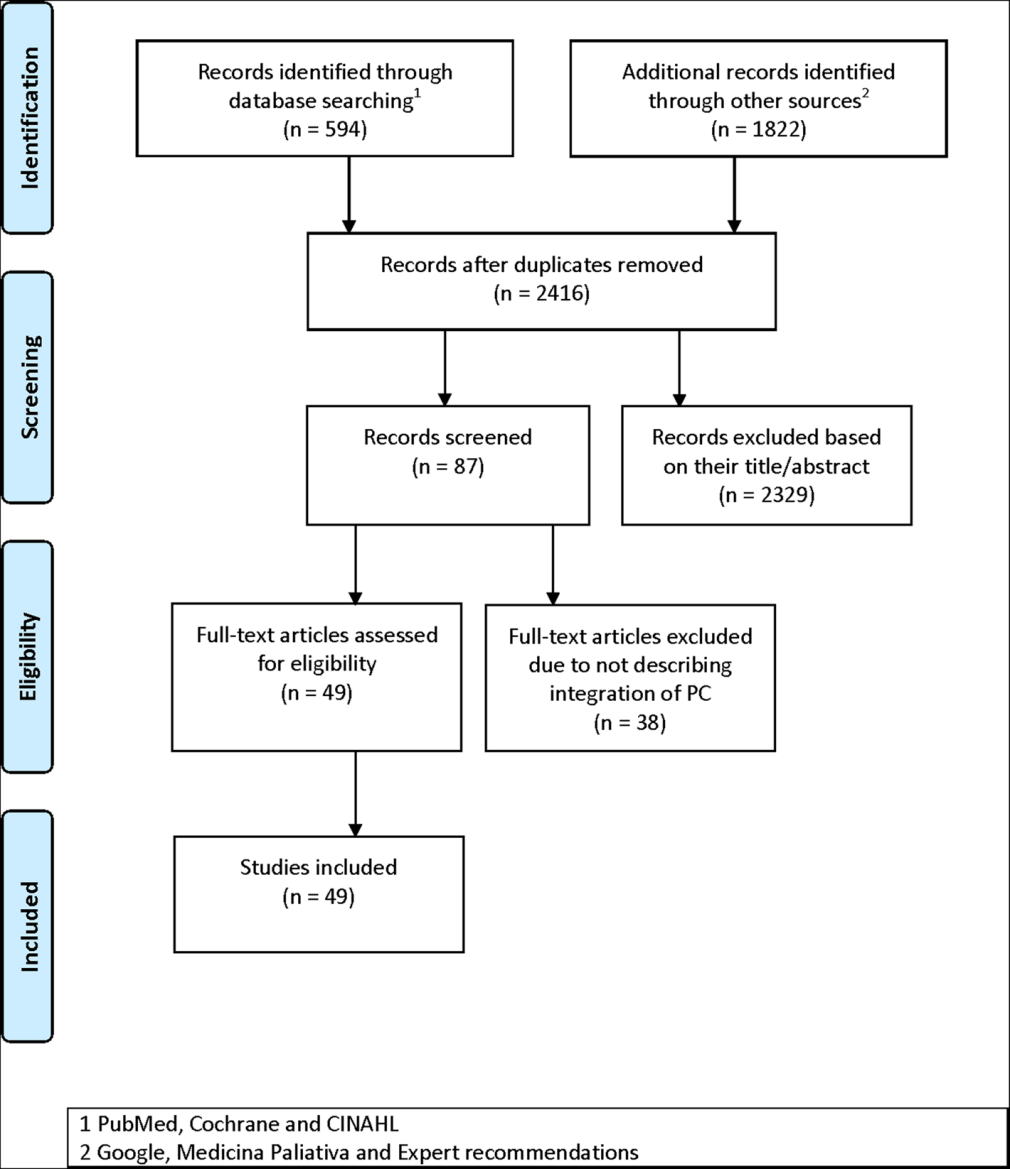
Flow diagram of the systematic review (modified from Moher et al. (2009))

After duplicates were removed, 2,416 documents were identified: PubMed 587 articles, Cochrane 2 documents, Cinahl 5 documents, the National Journal “Medicina Paliativa” (“Palliative Medicine”) 1020 articles, Google 800 documents, and two documents suggested by experts. After title and abstract screening 2329 records were excluded and after full text revision 49 documents were included for data extraction (Fig. 1).

In our review, two empirical models of IPC (Table 5), 12 guidelines and pathways in cancer and non-malignant diseases (Table 6) and 35 documents providing significant information relating to the Spanish context were identified (Table 7).

**Table 5:** Models of integrated Palliative Care (*n* = 2)

First author, and Year	Disease	Design	Quality assessment according to Hawker et al.	Intervention	Outcome measurements	Results/effectiveness of intervention
Vicente et al., 2010. [12]	Malignant and Non-malignant Disease	Retrospective and prospective cohort study	30	Influence of the Integrated Plan of PC^a^ of the Autonomous Community of Madrid in the medical activity of a hospital based PC^a^ unit.	Improvement in continuity of care, coordination amongst assistant bodies, increase in mean stay at the PCU^a^, increase in number of home deaths, etc.	PC home care improves continuity in care of patients. Transfers to intermediate stay care centers from 112 (14,7 %) to 144 (21,5 %) (*p* = 0,001) and deaths at home increased from 61 (8 %) to 97 (14,5 %) (*p* = 0,000). Median stay at the PCU ^a^ decreased from 7 to 6 days (*p* = 0,155).
Navarro et al., 2011.[11]	Advanced Chronic Disease	Observational, retrospective and descriptive study	26	EoLC^a^ of advanced chronic non-cancer patients identified by multidimensional evaluation and interdisciplinary teamwork in a medium and long term hospital.	General data, terminal criteria, diagnostic and prognostic information, development of advance directives, limiting levels of effort care, times from admission, risk of complicated bereavement.	Identification of advanced chronic non-cancer patients and their needs by interdisciplinary teamwork enabled indication for PC soon after admission (median 7 days, 15 days pure palliative treatment) and ensured appropriate care during their stay (prognostic to the family, increased from 65 % to 92 %; advance directives from 25 % to 96 %; adequacy level of care effort increased; Zarit score decreased, and risk of a complicated bereavement, 5 %.

^a^Abbreviations: *PC* palliative care, *PCU* palliative care unit, *EoLC* end of life care

**Table 6:** Clinical guidelines and pathways identified (*n* = 12)

Category	Reference	Date	Title	Disease	Setting	Emmanuel’s criteria^a^ *n* (%)	Recommendations based on…^b^
Guidelines in non-cancer	Aldasoro et al. [22]	2012	Necesidades en cuidados paliativos de las enfermedades no oncológicas. Un estudio cualitativo desde la perspectiva de profesionales, pacientes y personas cuidadoras (Needs in PC of the non oncologic diseases. A qualitative study from the professionals perspective, patients and carers)	Non cancer	All settings	4 (36 %)	Other options: Mixed methods corresponding to the “focused ethnography”
Pathways in non-cancer	Arnedillo et al. [23]	2012	Consenso sobre Atención Integral de las Agudizaciones de la Enfermedad Pulmonar Obstructiva Crónica ATINA-EPOC (Consensus on integrated care of acute exacerbations of chronic obstructive pulmonary disease ATINA-EPOC)	COPD	Not applicable	8 (73 %)	Consensus methods only
Pathways in non-cancer	Gómez-Batiste et al. [24]	2011	Proyecto NECPAL CCOMS-ICO. Identificación y atención integral-integrada de personas con enfermedades crónicas avanzadas en servicios de salud y sociales (NECPAL CCOMS-ICO Project. Identification and Integral-integrated attention of patients with advanced chronic diseases in health and social services)	Advanced chronic diseases	All settings	5 (45 %)	Systematic review and consensus methods
Pathway general approach	Agustín et al. [17]	2011	Manual para el manejo del paciente en Cuidados Paliativos en Urgencias Extrahospitalarias (Manual for patient management in PC in Extrahospital emergencies)	Cancer	Emergencies outside the hospitals	4 (36 %)	Consensus methods only
Guidelines general approach	SECPAL [15]	2010	Guía de Cuidados Paliativos (Palliative Care Guideline)	Cáncer and non cáncer	All settings	3 (27 %)	Unclear methods
Guidelines general approach	Colomer et al. [16]	2009	Unidad de Cuidados Paliativos: Estándares y recomendaciones (Palliative Care Unit: Standards and recommendations)	Cáncer and non cáncer	All settings	8 (73 %)	Systematic review and consensus methods
Guidelines general approach	Arrieta et al. [13]	2008	Guía de Práctica Clínica sobre Cuidados Paliativos (Clinical practical guideline on Palliative care)	Cancer and non cancer	All settings	10 (91 %)	Systematic review, consensus methods, evidence based and quality assessment
Guidelines general approach	González et al. [14]	2008	Guía de Cuidados Paliativos de la Comunidad de Madrid (Palliative Care guidelines of the Autonomous Community of Madrid)	Cancer and non cancer	All settings	5 (45 %)	Unclear methods
Pathways general approach	Cía et al. [18]	2007	Proceso asistencial integrado de Cuidados Paliativos (Palliative Care Integrated assistential Process)	Cancer and non cancer	Home and hospital settings	7 (63 %)	Systematic review and consensus methods
Guidelines in cancer	Carvajal et al. [19]	2006	Guía de recomendaciones clínicas: Cáncer colorrectal (Clinical recommendation guideline: Colon cancer)	Colorrectal cancer	All settings	4 (36 %)	Systematic review and consensus methods
Pathways in cancer	Naveira et al. [53]	2005	Cuidados paliativos en el enfermo oncologico. Documentos para la gestión integrada de procesos asistenciales relacionados con el cancer. Proyecto Oncoguias (Palliative Care for the oncologic patient. Documents for integrated management of assitential processes related to Cancer. “Oncoguías” Project)	Cancer	All settings	4 (36 %)	Unclear methods
Pathways general approach	Hernández et al. [17]	2004	Programa de cuidados domiciliarios en atención primaria (Home Care program in Primary attention)	Cancer and non cancer	Home setting	6 (56 %)	Unclear

^a^The 11 aspects assessed by “Emmanuel’s” are: discussion of illness limitations and prognosis; recommendations for conducting a whole patient assessment including the patient’s physical, social, psychological and spiritual issues, their family and community setting; recommendations for when to make these recommendations; recommendations on when PC should be integrated; assessment of the patient’s goals for care, continuous goal adjustment as the illness and the person’s disease progresses, PC interventions to reduce suffering, advance care planning, recommendations on involving a PC team, recommendations on PC at the last moments of life and recommendations on grief and bereavement [10]

^b^The guidelines/pathays’ recommendations were based on the following methods: 1) Systematic review, consensus methods, evidence based and quality assessment; 2) Systematic review and consensus methods; 3) Systematic review only; 4) Consensus methods only; 5) Unclear methods; 6) Other options

**Table 7:** Descriptive, strategic and analytical studies on PC integration in Spain (*n* = 35)

Category	Reference	Date	Documents titles	Type	Disease	Design	Setting	Total Hawker Score(1)
Descriptive documents (*n* = 4)	Alberola et al. [26]	2001	Modelos de Cuidados Paliativos en pacientes con cáncer (Pallitave Care models in cancer patients)	Models	Cancer	Models description	General University Hospital of Valencia; Oncology Catalan Institute, Barcelona; University Hospital “Dr Negrín”, Las Palmas; Clinic University Hospital of Valladolid	10
	Arrieta et al. [21]	2012	Cuidados paliativos. Proceso asistencial integrado Araba (Palliative Care. “Araba” Integrated assistential Process)	Models	Cancer and non-cancer	Model description	None	Not applicable
	Pascual N. [27]	2011	Modelos de atención a pacientes oncológicos terminales en Andalucía: una mirada sociológica (Models of oncologic terminal patients attention in Andalucia)	Models	Cancer	Multimode: Quantitative and qualitative tools	None	28
	Rubi et al. [25]	2005	Cuidados Paliativos en las enfermedades crónicas en fase avanzada. Situación actual y propuesta de organización asistencial (Palliative Care in advanced chronic respiratory disease. Status and assistential proposal for organisation)	Review and proposal	Advanced chronic respiratory disease	Narrative review	None	16
Strategic documents (*n* = 17)	Gómez-Batiste et al. [37]	2013	Identificación de personas con enfermedades crónicas avanzadas y neceisdad de cuidados paliativos en los servicios socio-sanitarios: herramienta NECPAL CCOMS-ICO© (Identification of people with chronic advanced diseases and need of palliative care in sociosanitary services: elaboration of the NECPAL CCOMS-ICO© tool)	Concrete situations	Advanced chronic diseases	Descriptive	None	15
	Miguez C. [33]	2010	Guía formativa del residente de oncología radioterápica. (Training guideline for resident of radiotherapic oncology)	Concrete situations	Radiotherapic oncology	Not applicable	None	Not applicable
	Castellanos et al. [35]	2007	Plan integral de atención sociosanitaria al deterioro cognitivo en Extremadura PIDEX (Integral plan of sociosanitary attention to cognitive impairment in Extremadura PIDEX)	Concrete situations	Cognitive impairment	Not applicable	None	Not applicable
	Herrera et al. [54]	2006	Primer nivel asistencial en Cuidados paliativos: Evolución del contenido de la cartera de servicios de atención primaria y criterios de derivación al nivel de soporte.(Primary Palliative Care: Development of the contents of the primary care services portfolio and criteria for referral according to complexity)	Concrete situations	Cancer and non-cancer	Not applicable	None	19
	Gómez-Batiste et al. [45]	2012	Proyecto de demonstración de la Organización Mundial de la Salud de Cataluña sobre implementación de cuidados paliativos: resultados cuantitativos y cualitativos en 20 años (The Catalonia World Health Organization demonstration project for palliative care implementation: quantitative and qualitative results at 20 years)	Assessment	Cancer and non-cancer diseases	Quantitative and qualitative	None	20
	Gómez-Batiste et al.	2007	Proyecto de demonstración de la Organización Mundial de la Salud de Cataluña en 15 años (Catalonia WHO palliative care demonstration project at 15 Years. 2005)	Assessment	Cancer and non-cancer	Unclear	None	20
	Gómez-Batiste et al. [39]	2006	Consumo de recursos y costes de los servicios de cuidados paliativos en España: un studio prospective multi-céntrico. (Resource consumption and costs of palliative care services in Spain: a multicenter prospective study)	Assessment	Cancer and non-cancer	Descriptive-observational, prospective, longitudinal Multicenter Study	None	22
	Cía et al. [28]	2008	Plan andaluz de Cuidados Paliativos (Andalusian plan for palliative care)	Regional Plan	Cancer and non-cancer	Not applicable	None	Not applicable
	Gago et al. [29]	2009	Estrategia de Cuidados Paliativos para Asturias (Palliative care strategy for Asturias)	Regional Plan	Cancer and non-cancer	Not applicable	None	Not applicable
	Amorín et al. [30]	2009	Programa de Cuidados Paliativos de Aragón (Palliative care strategy for Aragón)	Regional Plan	Cancer and non-cancer	Not applicable	None	Not applicable
	López et al. [31]	2010	Plan integral de Cuidados paliativos de la comunidad Valenciana (Palliative care integral plan for the Comunidad Valenciana)	Regional Plan	Cáncer and non-cancer	Not applicable	None	Not applicable
	Unknown	Unknown	Programa Integral Atención Paliativa Cantabria (Palliative integral attention programme for Cantabria)	Regional Plan	Cáncer and non-cancer	Not applicable	None	Not applicable
	Aguilera et al. [55]	2005	Plan integral de Cuidados Paliativos de la Comunidad de Madrid. 2005–2008 (Palliative Care integral plan for the Community of Madrid. 2005–2008)	Regional Plan	Cáncer and non-cancer	Not applicable	None	Not applicable
	Fernández et al. [33]	2007	Plan Integral de Cuidados Paliativos de la Comunidad Autónoma de la Región de Murcia 2006–2009 (Palliative Care integral plan for the Community of Murcia. 2006–2009)	Regional Plan	Cáncer and non-cancer	Not applicable	None	Not applicable
	García-Baquero et al. [14]	2010	Borrador del Plan estratégico de Cuidados paliativos de la Comunidad de Madrid, 2010–2014 (Draft of the Palliative Care strategic plan for the Community of Madrid,2010–2014)	Regional Plan	Cáncer and non-cancer	Not applicable	None	Not applicable
	Gobierno vasco [56]	2006	Plan de atención a pacientes en la fase final de la vida o Cuidados Paliativos de la Comunidad autónoma vasca (Plan of patients attention in end of life stage or Palliative Care in the Basque Autonomous región)	Regional Plan	Cancer and non-cancer	Not applicable	None	Not applicable
	Pascual et al. [2]	2011	Estrategia en Cuidados Paliativos del Sistema Nacional de Salud. Actualización 2010–2014 (Palliative Care Strategy of the National Health Service. Update 2010–2014)	National Plan	Cancer and non-cancer	Not applicable	None	Not applicable
Analytical studies (*n* = 14)	Rocafort et al. [57]	2006	Equipos de soporte de cuidados paliativos y dedicación de los equipos de atención primaria a pacientes en situación terminal en sus domicilios (Palliative care support teams and the commitment of primary care teams to terminally ill patients in their homes)	Observational	Cancer and non-cancer	Multicentre observational study.	None	19
	Ko W et al. [58]	2013	Awareness of general practitioners concerning cancer patients’ preferences for place of death: evidence from four European countries.	Observational	Cancer	Retrospective study	None	28
	Alonso et al. [59]	1997	Atención al paciente oncológico terminal en un distrito de atención primaria (Care for the terminal cancer patient in a primary care district)	Observational	Cancer	Unkown	None	Not applicable
	Canal et al. [60]	2005	Valoración de la implantación de un Programa Integral de Cuidados Paliativos. Visión retrospectiva 1995–1999 (Evaluation of the developement of a palliative car program. Review from 1995 to 1999)	Observational	Cancer	Retrospective and descriptive included in the ecologic category, by revising clinical records	Home care, inpatients units in long term and Inpatient Units in General Hospitals	18
	Simó et al. [61]	2006	Seguimiento de los pacientes atendidos conjuntamente por un equipo de Atención Primaria y su programa de Atención Domiciliaria y Equipos de Soporte (Follow-up of patients jointly cared for by a Primary Care unit, its Home Care program, and a Home Care Support Unit)	Observational	Multiple patologies: Dementia, neumologic diseases, neurologic diseases, cardiologic diseases, cancer, etcetera..	Descriptive and retrospective study	Urban basic health area of Terrasa (Barcelona)	21
	Riera et al. [62]	2008	Resultados de la evaluación de un instrumento de trabajo interdisciplinar: trayectoria clínica de la Unidad de Cuidados Paliativos (Implementation of an interdisciplinary working tool: Palliative Care Unit clinical pathway. Results of its evaluation)	Observational	Advanced or terminal cáncer	A retrospective study of clinical records	“Hospital de la Esperanza”, Barcelona, Spain.	22
	Costa et al. [63]	2012	Demencia avanzada y cuidados paliativos, características sociodemográficas y clínicas (Advanced dementia and palliative care, socio-demographic and clinical characteristics)	Observational	Advanced dementia	Observational descriptive	Antic Hospital St. Jaume i Sta Magdalena (Mataró, Barcelona)	22
	De Santiago et al. [64]	2012	Un nuevo equipo de soporte hospitalario en el departamento de oncología de un hospital universitario: evaluación de eficacia inicial y eficiencia. (A new palliative care consultation team at the oncology department of a university hospital: an assessment of initial efficiency and effectiveness)	Experimental	Cancer and non-cancer	Retrospective study	University of Navarre Clinic, Oncology department. Pamplona, Spain	22
	Alonso-Babarro et al. [65]	2013	La asociación entre la muerte del paciente, utilzación de recursos hospitalarios y disponibilidad de servicios domiciliarios para pacientes con cáncer. (The association between in-patient death, utilization of hospital resources and availability of palliative homecare for cancer patients)	Experimental	Cancer	Population-based study	Alcobendas-San-Sebastian de Los Reyes and Alcala de Henares districts	31
	Vega et al. [66]	2011	Atención sanitaria paliativa y de soporte de los equipos de atención primaria en el domicilio (Palliative and support care at home in primary care)	Experimental	Cancer and non-cancer	Descriptive	Five spanish sentinel networks between October 2007 and march 2008, in five Autonomous regions: Comunidad Valenciana, La Rioja, Castilla y León, Asturias and Extremadura	16
	Prades et al. [67]	2011	Tratamiento multidisciplinar en cancer en España, o cuando la función crea el órgano: estudio de entrevista cualitativa. (Multidisciplinary cancer care in Spain, or when the function creates the organ: qualitative interview study)	Experimental	Cancer	Qualitative interview study with semi-structured, one-to-one interviews	Most populated regions of Spain, namely, Andalusia, Catalonia, Madrid, Galicia and Valencia	19
	Colchero et al. [68]	2009	Atención en pacientes oncológicos terminals en un distrito de atención primaria (Care of terminally ill oncology patients in an urban primary care district)	Experimental	Cancer	Transversal descriptive study	2 hospital areas in the Seville’s primary attention district, 32 centres and 278 patients	11
	Agra et al. [69]	2003	Relación de la calidad de vida con diferentes modelos de atención domiciliaria en enfermos oncológicos terminales de un área sanitaria de Madrid (Relationship between quality of life and various models of home care in terminal oncology patients from a health area of Madrid)	Experimental	Cancer	A quasi-experimental prospective study	Area 4 of the “Imsalud” in Madrid	25
	Rihuete et al. [70]	2005	Atención integral al paciente oncológico y su familia desde una intervención multidisciplinar (Integral attention to oncology patients and their relatives from a multidisciplinary team)	Experimental	Cancer	Retrospective analysis of social interventions and a new methodology of proactive intervention, employing multidisciplinary clinic social sessions. And finally both interventions were compared	Unit of Oncology in the University of Salamanca	14

(1) Protocol of the Hawker et al. appraisal tool [9] punctuates from 1 (very poor) to 4 (good) the following aspects: 1. Abstract and title: Did they provide a clear description of the study?; 2. Introduction and aims: Was there a good background and clear statement of the aims of the research?; 3. Method and data: Is the method appropriate and clearly explained?; 4. Sampling: Was the sampling strategy appropriate to address the aims?; 5. Data analysis: Was the description of the data analysis sufficiently rigorous?; 6. Ethics and bias: Have ethical issues been addressed, and what has necessary ethical approval gained? Has the relationship between researchers and participants been adequately considered?; 7. Results: Is there a clear statement of the findings?; 8. Transferability or generalizability: Are the findings of this study transferable (generalizable) to a wider population?; 9. Implications and usefulness: How important are these findings to policy and practice?

### Models

Two models were identified: “Atención a pacientes crónicos avanzados no oncológicos con necesidad de cuidados al final de la vida en un hospital de media y larga estancia” (End-of-life care of advanced chronic non-cancer patients in a medium and long term hospital) [11] and the document “Influencia del Plan Integral de Cuidados Paliativos de la Comunidad de Madrid” (Influence of the Integrated Plan of Palliative Care of the Autonomous Community of Madrid in the medical activity of a hospital based palliative care unit) [12].

These two models, both published in 2011 and applying to hospital settings, achieved a high quality score (Hawker et al.,) [9]. Both were observational studies, the first of which addressed non-cancer patients suffering from advanced chronic disease, identified and evaluated through an exhaustive multidimensional study and interdisciplinary teamwork in a long stay hospital delivering end-of-life care [11]. The second, considered both cancer and non-cancer disease and concluded that the integration of a Home Care team within a PC unit improves continuity of care and coordination between levels of healthcare [12] (Table 5).

### Guidelines and pathways

#### Guidelines and pathways on both malignant and non-malignant diseases

Four clinical guidelines for cancer and non-cancer were identified. The “Guía de Práctica Clínica sobre Cuidados Paliativos” (Clinical Practice Guideline on Palliative Care) [13], published in 2008, matched 10 out of 11 Emmanuel criteria [10]. This guideline is in line with the other three identified in that they all included recommendations about PC interventions to reduce suffering [14–16]. Except for “Guía de Cuidados Paliativos” (Palliative Care Guideline), the other three concurred in three other criteria: discussion of illness limitations and prognosis; recommendations for conducting a whole patient assessment including their family and their community setting; and recommendations on when PC should be integrated (Table 6).

Three pathways in cancer and non-cancer were found. One pathway referred to out-of-hospital emergencies [17], another related to home settings [18], and finally, one applied to both [19]. The three pathways contain recommendations on when PC should be integrated; suggestions to intervene to reduce suffering as needed; and recommendations on care during the last hours of living (Table 6).

### Guidelines and pathways on cancer

One clinical guideline, “Guía de recomendaciones clínicas: cancer colorectal” (Guideline of clinical recommendations: colon cancer) [20] and one pathway, “Cuidados paliativos en el enfermo oncológico, documentos para la gestión integrada de procesos asistenciales relacionados con el cáncer, Proyecto Oncoguías” (Palliative care in the oncologic patient, documents for integrated management of care processes related to cancer, Oncoguías Project) [21], addressed cancer patients. Four out of 11 Emmanuel’s criteria [10] appeared for both and were applicable to all settings. The “Guía de recomendaciones clínicas: cancer colorectal” (Guideline of clinical recommendations: colon cancer) [20] used systematic review and consensus methods, so was considered good quality. Commonly, these two documents include recommendations for conducting a whole patient assessment including patient’s physical, social, psychological, and spiritual issues within their family and community setting, and recommendations on grief and bereavement care (Table 6).

### Guidelines and pathways on non-cancer

One clinical guideline [22] and two pathways (one relating to Chronic Obstructive Pulmonary Disease (COPD) and the other, general chronic advanced illness) were identified [23, 24]. They were published between 2011–2012 and apply to all settings. These three rated well in terms of IPC against Emmanuel’s criteria [10] (see Table 6). The two pathways share recommendations for conducting a whole patient assessment, including their family and community setting; assessment of the patient’s goals of care; continuous goal adjustment as the disease progresses; and the presence of the advance care planning criterion (Table 6).

### Analytical comparison of all guidelines and pathways

Five of the documents (42 %) were above the average in Emmanuel’s criteria [10] and seven (58 %) below. All exceeded the initial filter of achieving at least two out of the 11 criteria.

With regard to the recommendations appearing, there exist large variations. The most reported recommendations are: conduction of a whole patient assessment including patient’s physical, social, psychological, and spiritual issues in the context of their family and their community setting; recommendations on when PC should be integrated; and suggestions to intervene to reduce suffering as needed. These recommendations were found in three quarters of the selected documents. Conversely, the least cited recommendations are those related to the timing of assessments and to continuous goal adjustment as illness progresses. (Additional file 1: Table S1).

### Descriptive, strategic and analytical documents

Strategic documents (*n* = 17), descriptive documents (*n* = 4), and analytical studies (*n* = 14) were found (Table 7).

### Descriptive documents

Amongst these, there are papers describing diverse programmes with fruitful collaborations between PC teams and oncology departments in a narrative review of patients suffering from advanced chronic respiratory disease [25]; descriptions of an integrated PC process [21, 26] and a description of how care provision for terminal cancer patients was organised in Andalucia in the year 2000 [27].

### Strategic documents

Nine of the strategic documents (53 %) are produced at a regional level (Andalucía [28], Asturias [29], Aragón [30], Comunidad Valenciana [31], Cantabria, Madrid [14, 32], Murcia [33] and the Basque Country [34]), and one (5 %) at a National level. This latter is an update (2010–2014) for the Strategy in Palliative Care of the National Health System released by the Health, Social Policies and Equality Ministry [2].

These strategic documents are particularly important in Spain due to the nature of the Spanish National Health System, as it is a decentralised governmental system where healthcare regions have considerable control on the delivery of health care.

Four other documents (24 %) address integration of PC in concrete situations; radiotherapy [35], cognitive impairment [36], advanced chronic disease [37] and primary care [38].

Finally, a set of three documents (18 %) have been categorized as assessment documents; two of these evaluated resource consumption and cost effectiveness [39, 40], and the other, assessment of PC implementation by a WHO demonstration project [41].

### Analytical studies

These documents were identified within PUBMED (*n* = 8, 57 %) or the Spanish journal Medicina Paliativa (Palliative Medicine) (*n* = 6, 43 %) and included 7 observational and 7 experimental, intervention studies.

Analytical studies mainly focused on integration of PC into primary care, resource utilisation, focusing on cancer, dementia, and other non-cancer conditions. In contrast to the models (noted above), these scored moderately against the Hawker et al. tool, scoring under 22 out of a possible 36.

The observational studies include descriptive and retrospective designs describing and analysing clinical records, whereas the experimental studies involved pro-actively engaging with patients and PC professionals as study participants. These adopted diverse study designs including: retrospective, population-based, descriptive, structured and semi-structured interviews, quasi-experimental prospective and prospective cohort studies.

The majority of these addressed cancer (*n* = 9, 64 %), a few refer to cancer and non-cancer diseases (*n* = 4, 29 %), and just one (7 %) considered non-cancer alone. The settings to which these studies apply varied from PC units in long term and general hospitals, home care situations and wider health regions. All these studies conclude that IPC have positive impacts on the quality of life of patients and their families, improves patient perception of their own health condition, and reduces inpatient deaths and hospitalizations in the last months of life.

## Discussion

A total of 49 documents including models, clinical guidelines and pathways, and other strategic, descriptive and analytical documents have been identified. The majority of guidelines and pathways scored well against Emmanuel’s criteria [10] in terms of PC integration. Strategic, analytical and descriptive studies evaluated with Hawker et al. tool [9] show that for 15 of these (43 %), the score was above half of the total attainable points.

Spain has included PC into guidelines and pathways on cancer in a good theoretical level of integrated PC (as assessed by Emmanuel’s criteria) meaning that key point elements for conducting IPC for cancer patients are included within guidelines and pathways, at the time that quality was considered high.

In contrast, the number identified for chronic advanced diseases suggest that Spain is at an early stage if we take chronic obstructive pulmonary disease or chronic heart failure (CHF) as exemplars. Only a few of these could be considered robust in terms of developmental methods and level of evidence, as they are supported mainly on consensus processes, and further evaluation should be applied to evaluate quality. Just one guideline: “Guía de Práctica Clínica sobre Cuidados Paliativos” (Clinical Practice Guideline on Palliative Care) can be considered of high quality matching 10 out of 11 criteria on the Emmanuel scale [10].

Another two documents, the guideline “Unidad de Cuidados Paliativos: Estándares y recomendaciones” (Palliative Care Unit: standards and recommendations) [16] and the pathway “Consenso sobre Atención Integral de las Agudizaciones de la Enfermedad Pulmonar Obstructiva Crónica ATINA-EPOC” (Consensus on integrated care of acute exacerbations of chronic obstructive pulmonary disease ATINA-EPOC) [23], are good examples as contain 8 out of 11 Emmanuel’s key recommendations. These –jointly with the previously indicated guideline-, place an emphasis in organisational and clinical aspects bearing in mind the importance of coordination, dialogue and constant relationship between units and assistance levels. All aiming to guarantee continuity of care between all agents involved in the PC process.

Overall, this review demonstrates some progress in IPC by Spanish health providers and policy makers and suggests general agreement on the need for the integration of palliative care in service provision. This is supported by the existence of strategic documents and it seems that planning for IPC is a major concern for the Spanish Public Health System and its different regional services. That said, implementation plans should be developed beyond the theory [42, 43].

The National Health System in Spain included the enhancement of attention to PC as one of the strategic priorities within the Quality Plan of the National Health System [44]. On this basis, the challenge of integrating multidisciplinary PC teams, attention to continuity of care, and coordination between different levels is already being addressed [3].

Other studies are proceeding similarly, for example by reviewing the current situation of IPC in the region of Catalonia and moreover, identifying possible areas of improvement [40, 45]. The focus of this paper is Spain, as a whole, and suggests the need for further research on the topic, in order to improve the quality of life and palliative care provision for patients and their families.

International studies have recently investigated IPC at an European level, seeking and analysing guidelines and pathways for adult cancer patients [46], for COPD or CHF patients [47], and another non-published paper seeking empirically-tested models both in cancer and chronic diseases [48]. Similarly to our study, both in cancer and non-cancer guidelines and pathways, most frequent key components of IPC (according to Emmanuel’s list) are: holistic approach and suggestions to intervene to reduce suffering as needed [46, 47]. Interestingly, it is noticeable the frequency difference of grief and bereavement contents in guidelines and pathways for non-cancer patients between Spain (60 %) and European countries (21 %) [47].

Lately, a full and varied body of research has been published including several reviews on IPC from diverse perspectives. Amongst them, there is a review of evidence reporting the positive impact of engaging communities in end-of-life care [49]. Secondly, an integrative review addresses paediatric PC and psychosocial support in oncology settings, revealing a set of issues to develop comprehensive psychosocial PC standards [50]. A study on barriers/opportunities to IPC in the United States from a public health perspective highlights the lack of education/training, inadequate size of trained workforce and several policy barriers such as regulatory barriers, lack of funding for research, problems in reimbursement and a fragmented healthcare system [51]. Finally, a narrative synthesis reviewing themes that facilitate and hinder collaboration between hospital-based generalist PC professionals and in-patient specialist PC professionals, finds out five themes essential to either enhancing or worsening effective collaboration: model of care, professional onus, expertise and trust, skill building and specialist PC operations [52].

Readers should be aware that the number of sources used in this review is limited (PUBMED, Cochrane Library, Cinahl, Google, Medicina Paliativa) although we have considered the most appropriate available for our context. PUBMED includes all the most important journals, Cinahl is the most important nursing database, Medicina Paliativa is the only PC Spanish journal, Cochrane Library was included to explore secondary information and Google brings Scielo’s articles as well as grey literature. It must be also acknowledged that the Google hits considered stopped when reaching 800 hits as Google itself orders and allocates in first positions most relevant documents, which does not mean that other results could have been considered.

The concept of IPC itself remains a developing concept with all its implications in terms of robust, published research. This is a first work in this area relating to Spanish-speaking countries. We suggest that others could benefit from this and may seek to replicate our methods to investigate the situation of IPC in their own countries literature.

A beneficial approach for the future might be to test whether models, plans, guidelines and pathways, as outlined above, have been used with positive effect and demonstrable service improvements for patients and families in receipt of palliative care. These aspects could allow to compare and implement a kind of benchmarking which could be useful for policy makers and managers among others.

## Conclusions

The existence of scarce implemented IPC models, the number and quality of clinical guidelines and pathways, and the large amount of other relevant documents addressing IPC seem to demonstrate that IPC is at an incipient development stage in Spain.

Documents from a strategic, descriptive and analytical perspective, overall point out the achievements in terms of policy makers and health providers agreements and contextualise a potential environment.

From our review, it can be said that first steps towards IPC in Spain have been made, but the literature lacks sufficient evidence about implementation and therefore highlights that much work remains to be done.

### Ethics approval and consent to participate

There are no human participants involved.

### Consent for publication

There are no any individual person’s data.

### Availability of data and materials

Not applicable.

## Additional file

Additional file 1: Display of the Emmanuel’s criteria per study. (DOCX 25 kb)

## Abbreviations

CHF: chronic heart failure
COPD: chronic obstructive pulmonary disease
IPC: integrated palliative care
PC: palliative care
WHO: World Health Organisation
