# Supplementary material for: Integrated palliative care in the Spanish context: a systematic review of the literature
Source: BMC Palliat Care. 2016 May 13;15:49. doi: 10.1186/s12904-016-0120-9 (PMC4865984; doi:10.1186/s12904-016-0120-9)
Supplement: Additional file 1: — Display of the Emmanuel’s criteria per study. (DOCX 25 kb) [file 12904_2016_120_MOESM1_ESM.docx]

| Category | Reference | Date | Title | Emmanuel´s criteria  (n, %) [10] | List of Key Emmanuel´s recommnedations included |
| --- | --- | --- | --- | --- | --- |
| Guidelines in non-cancer | Aldasoro et al. [19] | 2012 | Necesidades en cuidados paliativos de las enfermedades no oncológicas. Un estudio cualitativo desde la perspectiva de profesionales, pacientes y personas cuidadoras ( Needs in PC of the non oncologic diseases. A qualitative study from the professionals perspective, patients and carers) | 4 (36%) | 1. Discussion of illness limitations and prognosis  2. Recommendations on when PC should be integrated  3. Advance care planning  4. Recommendations on involving a PC team |
| Pathways in non-cancer | Arnedillo et al. [20] | 2012 | Consenso sobre Atención Integral de las Agudizaciones de la Enfermedad Pulmonar Obstructiva Crónica ATINA-EPOC (Consensus on integrated care of acute exacerbations of chronic obstructive pulmonary disease ATINA-EPOC) | 8 (73%) | 1. Discussion of illness limitations and prognosis  2. Recommendations for conducting a whole patient assessment including the patient´s physical, social, psychological and spiritual issues, their family and community setting  3. Recommendations for when to make these recommendations  4. Recommendations on when PC should be integrated  5. Assessment of the patient´s goals for care  6. Continuous goal adjustment as the illness and the person´s disease progresses.  7. PC interventions to reduce suffering  8. Advance care planning |
| Pathways in non-cancer | Gómez-Batiste et al. [21] | 2011 | Proyecto NECPAL CCOMS-ICO. Identificación y atención integral-integrada de personas con enfermedades crónicas avanzadas en servicios de salud y sociales  (NECPAL CCOMS-ICO Project. Identification and Integral-integrated attention of patients with advanced chronic diseases in health and social services) | 6 (55%) | 1. Recommendations for conducting a whole patient assessment including the patient´s physical, social, psychological and spiritual issues, their family and community setting  2. Assessment of the patient´s goals for care  3. Continuous goal adjustment as the illness and the person´s disease progresses  4. Advance care planning  5. Recommendations on involving a PC team  6. Recommendations on grief and bereavement |
| Pathway general approach | Agustín et al. [14] | 2011 | Manual para el manejo del paciente en Cuidados Paliativos en Urgencias Extrahospitalarias (Manual for patient management in PC in Extrahospital emergencies) | 4 (36%) | 1. Discussion of illness limitations and prognosis  2. Recommendations on when PC should be integrated  3. PC interventions to reduce suffering  4. Recommendations on PC at the last moments of life |
| Guidelines general approach | SECPAL [44] | 2010 | Guía de Cuidados Paliativos (Palliative Care Guideline) | 3 (27%) | 1. PC interventions to reduce suffering  2. Recommendations on PC at the last moments of life  3. Recommendations on grief and bereavement |
| Guidelines general approach | Colomer et al. [45] | 2009 | Unidad de Cuidados Paliativos: Estándares y recomendaciones (Palliative Care Unit: Standards and recommendations) | 8 (73%) | 1. Discussion of illness limitations and prognosis  2. Recommendations for conducting a whole patient assessment including the patient´s physical, social, psychological and spiritual issues, their family and community setting  3. Recommendations on when PC should be integrated  4. Assessment of the patient´s goals for care  5. PC interventions to reduce suffering  6. Advance care planning  7. Recommendations on involving a PC team  8. Recommendations on grief and bereavement |
| Guidelines general approach | Arrieta et al. [46] | 2008 | Guía de Práctica Clínica sobre Cuidados Paliativos (Clinical practical guideline on Palliative care) | 10 (91%) | 1. Discussion of illness limitations and prognosis  2. Recommendations for conducting a whole patient assessment including the patient´s physical, social, psychological and spiritual issues, their family and community setting  3. Recommendations for when to make these recommendations  4. Recommendations on when PC should be integrated  5. Assessment of the patient´s goals for care  6. PC interventions to reduce suffering  7. Advance care planning  8. Recommendations on involving a PC team  9. Recommendations on PC at the last moments of life  10. Recommendations on grief and bereavement |
| Guidelines general approach | González et al. [29] | 2008 | Guía de Cuidados Paliativos de la Comunidad de Madrid (Palliative Care guidelines of the Autonomous Community of Madrid) | 5 (45%) | 1. Discussion of illness limitations and prognosis  2. Recommendations for conducting a whole patient assessment including the patient´s physical, social, psychological and spiritual issues, their family and community setting  3. Recommendations on when PC should be integrated  4. PC interventions to reduce suffering  5. Recommendations on PC at the last moments of life |
| Pathways general approach | Cía et al. [15] | 2007 | Proceso asistencial integrado de Cuidados Paliativos (Palliative Care Integrated assistential Process) | 7 (63%) | 1. Recommendations for conducting a whole patient assessment including the patient´s physical, social, psychological and spiritual issues, their family and community setting  2. Recommendations on when PC should be integrated  3. PC interventions to reduce suffering  4. Advance care planning  5. Recommendations on involving a PC team  6. Recommendations on PC at the last moments of life  7. Recommendations on grief and bereavement |
| Guidelines in cancer | Carvajal et al. [16] | 2006 | Guía de recomendaciones clínicas: Cáncer colorrectal (Clinical recommendation guideline: Colon cancer) | 4 (36%) | 1. Recommendations for conducting a whole patient assessment including the patient´s physical, social, psychological and spiritual issues, their family and community setting  2. Recommendations on when PC should be integrated  3. Continuous goal adjustment as the illness and the person´s disease  4. Recommendations on grief and bereavement |
| Pathways in cancer | Naveira et al. [46] | 2005 | Cuidados paliativos en el enfermo oncologico. Documentos para la gestión integrada de procesos asistenciales relacionados con el cancer. Proyecto Oncoguias (Palliative Care for the oncologic patient. Documents for integrated management of assitential processes related to Cancer. "Oncoguías" Project) | 4 (36%) | 1. Recommendations for conducting a whole patient assessment including the patient´s physical, social, psychological and spiritual issues, their family and community setting  2. PC interventions to reduce suffering  3. Recommendations on involving a PC team  4. Recommendations on grief and bereavement |
| Pathways general approach | Hernández et al. [14] | 2004 | Programa de cuidados domiciliarios en atención primaria (Home Care program in Primary attention) | 6 (56%) | 1. Recommendations for conducting a whole patient assessment including the patient´s physical, social, psychological and spiritual issues, their family and community setting  2. Recommendations on when PC should be integrated  3. PC interventions to reduce suffering  4. Recommendations on involving a PC team  5. Recommendations on PC at the last moments of life  6. Recommendations on grief and bereavement |
